# Practice nurses' workload, career intentions and the impact of professional isolation: A cross-sectional survey

**DOI:** 10.1186/1472-6955-9-2

**Published:** 2010-01-25

**Authors:** Catherine A O'Donnell, Hussein Jabareen, Graham CM Watt

**Affiliations:** 1 General Practice & Primary Care, Division of Community-based Sciences, Faculty of Medicine, University of Glasgow, 1 Horselethill Road, Glasgow G12 9LX, UK

## Abstract

**Background:**

Practice nurses have a key role within UK general practice, especially since the 2004 GMS contract. This study aimed to describe that role, identify how professionally supported they felt and their career intentions. An additional aim was to explore whether they felt isolated and identify contributory factors.

**Methods:**

A cross-sectional questionnaire survey in one large urban Scottish Health Board, targeted all practice nurses (n = 329). Domains included demographics, workload, training and professional support. Following univariate descriptive statistics, associations between categorical variables were tested using the chi-square test or chi-square test for trend; associations between dichotomous variables were tested using Fisher's Exact test. Variables significantly associated with isolation were entered into a binary logistic regression model using backwards elimination.

**Results:**

There were 200 responses (61.0% response rate). Most respondents were aged 40 or over and were practice nurses for a median of 10 years. Commonest clinical activities were coronary heart disease management, cervical cytology, diabetes and the management of chronic obstructive pulmonary disease. Although most had a Personal Development Plan and a recent appraisal, 103 (52.3%) felt isolated at least sometimes; 30 (15.5%) intended leaving practice nursing within 5 years.

Isolated nurses worked in practices with smaller list sizes (p = 0.024) and nursing teams (p = 0.003); were less likely to have someone they could discuss a clinical/professional (p = 0.002) or personal (p < 0.001) problem with; used their training and qualifications less (p < 0.001); had less productive appraisals (p < 0.001); and were less likely to intend staying in practice nursing (p = 0.009). Logistic regression analysis showed that nurses working alone or in teams of two were 6-fold and 3.5-fold more likely to feel isolated. Using qualifications and training to the full, having productive appraisals and planning to remain in practice nursing all mitigated against feeling isolated.

**Conclusions:**

A significant proportion of practice nurses reported feeling isolated, at least some of the time. They were more likely to be in small practices and more likely to be considering leaving practice nursing. Factors contributing to their isolation were generally located within the practice environment. Providing support to these nurses within their practice setting may help alleviate the feelings of isolation, and could reduce the number considering leaving practice nursing.

## Background

Practice nurses are an integral part of general practice/family medicine teams in the UK, with a role which encompasses general treatment room duties, nursing duties and chronic disease management [1].

In 2004, a new General Medical Services contract was introduced in the UK. Unlike previous contracts, this is held at practice-level, not with individual general practitioners (family practitioners) [2]. Another key development was the introduction of the Quality and Outcomes Framework (QOF), a pay-for-performance measure covering both clinical and organisational areas of work [3]. Within the clinical domains, there is a focus on chronic diseases with points awarded for care in areas such as coronary heart disease, diabetes and asthma and it is estimated that practices can now earn up to one-third of their income from QOF payments, by meeting these targets [4]. Practice nurses have played a key role in the achievement of QOF points [5-7], as predicted when the contract was implemented [3,8]. However, while the evidence suggests that practice nurses are embracing these new roles, there have been negative consequences too. Nurses complain that their workload has increased dramatically, that adherence to "box-ticking" for the QOF impacts on the holistic nature of the nurse-patient consultation and that their remuneration has been less than expected, given the financial gains for practices [5-7].

Practice nurses are employees of the doctors in the practice where they work. While there are advantages to this in terms of the cohesiveness of practice teams, disadvantages include the exclusion of practice nurses from many strategic documents, including *Agenda for Change *which outlined new terms and conditions of employment for non-medical NHS staff [9], and the lack of nationally recognised terms and conditions for employment [10]. Practice nurses, particularly those working in small practices, may also be more likely to work alone with fewer opportunities for inter-professional contact, reflecting the situation faced by doctors working in small practices. However, while the impact of isolation has been the focus of attention when it affects doctors [11], there has been little or no attention paid to professional isolation as it impacts on practice nurses.

These developments need to be considered in the wider context of nursing recruitment and retention. Recruitment and retention of staff presents challenges for both nursing and medicine, in the UK and abroad [12-16]. While many studies have focussed on secondary care nursing, primary care is faced with similar problems [17,18]. Buchan identified that, by 2010, one in four nurses would be aged 50 or more, with general practice nursing particularly affected [17]. Other factors associated with problems in the recruitment and retention of nurses include job dissatisfaction [12] and perceived work ability, a concept which includes commitment to education and training, employment history, relationships with colleagues and managerial support [16].

In an attempt to explore some of these issues, and to inform the development of later qualitative work exploring nurses' views of their role post-GMS contract, we conducted a questionnaire survey in one large urban Health Board area in Scotland. Conducted late in 2005, we wished to describe the role that practice nurses were undertaking post-GMS contract, to find out how professionally supported they felt in their work and to identify their career intentions. In particular, we used this as an opportunity to explore whether or not nurses felt isolated in their daily role and what factors may contribute to that. This work was conducted in collaboration with the Health Board, who wished to use the findings of the questionnaire to develop support structures for practice nurses and to inform workforce planning.

## Methods

### Study design and setting

The study was a descriptive cross-sectional survey of practice nurses working in general practice within a large, urban Health Board, using a self-completion postal questionnaire. It was conducted in collaboration with the Health Board's Primary Care Division practice nurse advisor and the workforce planning project manager.

### Study population and questionnaire distribution

The target population was all 329 practice nurses working within the Health Board in 2005. The practice nurse advisor distributed the questionnaire on our behalf; completed questionnaires were returned to the research team. The Local Research Ethics Committee requested that no nurse or practice identifier be included on the questionnaire, thus a blanket reminder was sent out 21 days after the initial questionnaire, again through the practice nurse advisor. Completion of the questionnaire was taken to mean the nurses consented to participate in the survey, i.e. implied consent.

### Questionnaire design

Questionnaire items were derived from three sources: a literature review on the role of practice nurses; discussions with nurses in management positions within the Health Board; and a previous questionnaire conducted by the practice nurse advisor in early 2004. The literature review covered a range of areas, including the development of the practice nurse role in primary care; practice nurse workload; policy drivers contributing to the development of the practice nurse role (for example, *Liberating the Talents *[19] and *Agenda for Change *[9]; and literature on skill mix and role development, including work by Sibbald et al on skill mix [20] and Daly and Carnwell's work on developing a framework for nursing roles [21]. Items from the previous questionnaire on nursing activities and training were also included.

The final questionnaire covered six domains, with 90 items (see Additional File 1). The domains were personal demographics; practice structure; professional and educational qualifications and career intentions; workload and clinical roles; training and continuing professional development; access to professional support.

Most items were categorical variables, some dichotomous. At the end of the questionnaire respondents were given the opportunity to add any further comments regarding their role and support issues. Before distribution, the questionnaire was shown to colleagues and nurses undertaking the Master in Primary Care within General Practice and Primary Care to assess the ease of completion and validity of the questionnaire.

### Data entry and analysis

Responses were entered into SPSS 11.5 by HJ. A 10% sample was double entered by a departmental secretary to check for data quality and consistency. No major issues in the accuracy of data entry were detected.

Descriptive univariate analyses were conducted using frequency tables; not all practice nurses provided an answer for every question, so the results are presented as the number and frequency (%) of responses. Continuous variables were not normally distributed, therefore median and inter-quartile ranges were reported and comparisons analysed by the Mann-Whitney U test. Bivariate analysis was used to further explore the association between isolation and a range of variables. Associations between categorical variables were tested using the chi-square test or, where one variable was ordered, the chi-square test for trend. Fisher's Exact test was used to examine associations between dichotomous variables [22]. Variables that, on bivariate analyses, were significantly associated with isolation (p < 0.05) were entered into a binary logistic regression model using backwards elimination [23].

### Ethical approval

The study was approved by the NHS Greater Glasgow Primary Care Research Ethics Committee (REC Reference Number: 05/S0706/30).

## Results

### Demographics and practice characteristics

A response rate of 61% was obtained (200/329 nurses). All respondents were female. About half (49.0%) were aged 40-49 years; 29.0% were 50 or more (Table 1). The majority were Grade G nurses and were Registered General Nurses or State Registered Nurses. However, most had multiple qualifications: 80 (40.0%) had 2 qualifications; 54 (27.0%) had 3 or more. These included district nursing, specialist nurses in general practice and the practice nurse certificate (Table 1).

**Table 1:** Description of respondents.

	Number (%)
**Age categories (years)**	
20 - 39	43 (21.7)
40 - 49	97 (49.0)
50 and above	58 (29.3)
	
**Grade**	
D, E or F	15 (7.8)
G	142 (73.6)
H	36 (18.7)
	
**Qualifications^a^**	
Registered General Nurse/State Registered Nurse	192 (96.0)
Enrolled Nurse	19 (9.5)
Undergraduate Nursing Degree	45 (22.5)
State Certified Midwife/State Midwife	50 (25.0)
Registered Mental Health Nurse	7 (3.5)
District Nurse	20 (10.0)
Health Visitor	3 (1.5)
Specialist Nurse in General Practice	40 (20.0)
Practice Nurse Certificate	24 (12.0)
Masters Degree	7 (3.5)
	
**Practice list size**	
Up to 2000 patients	17 (8.9)
2001 - 4000	49 (25.7)
4001 - 6000	53 (27.7)
6001 - 8000	27 (13.5)
8001 - 10,000	28 (14.7)
Over 10,000 patients	17 (8.9)
	
**Number of practice nurses in the team**	
1	61 (30.8)
2	85 (42.9)
3 or more	52 (26.3)

a. Adds to more than 200, as multiple responses were permitted.

Respondents had worked as practice nurses for 0.5 to 24.0 years, median = 10.0 years (interquartile range (IQR): 5.0 - 15.0 years). The length of service in their present practice ranged from 0.5 to 24.0 years, median = 7.0 years (IQR: 3.0 - 12.0 years).

The majority (102, 53.4%) worked in practices with between 2000 and 6000 patients, although about one-fifth worked in very small (<2000 patients) or very large (>10,000 patients) practices (Table 1). Reflecting this, 43.0% worked with one other nurse, 26.0% worked with two other nursing colleagues, but 31.0% worked alone (Table 1). Almost all respondents (192, 98.0%) worked in clinics with an appointment system with a median of 26 appointment slots per day (IQR: 20.0 - 33.8). The median length per appointment was 15.0 minutes (IQR: 10.0 - 15.0 minutes).

### Workload and training

Nurses were asked about their current clinical activities within the practice (Table 2). Amongst those who responded to these questions (approximately half of the total sample), the most common activities were coronary heart disease (CHD) management (92.0%), cervical cytology (91.7%), travel immunizations (89.8%) and health promotion (87.7%). The next most common activities involved chronic disease management (stroke (85.1%), asthma (84.0%), diabetes (84.0%) and chronic obstructive pulmonary disease (COPD) (80.2%)). The least common activities were childhood immunizations (29.9%) and assisting with minor surgery (23.6%). Nurses had received specialist training in all clinical areas, particularly cervical cytology (92.6%), diabetes (88.4%), CHD (86.2%) and asthma (83.0%). The areas where least training had been received were men's health (24.4%) and assisting with minor surgery (23.0%). Reflecting this, 64.2% of respondents wanted more training in men's health; however the biggest request was for more training in treating minor illness (66.9% of respondents).

**Table 2:** Workload and training needs amongst practice nurses (Number answering yes/Total number of respondents (%)).

	Currently carrying out activity	Received specialised training in the past	Would like more specialised training
Assisting with minor surgery	25/106 (23.6)	34/148 (23.0)	31/110 (28.2)
Childhood immunizations	32/107 (29.9)	61/154 (39.6)	46/116 (39.7)
Clinical leadership & managing other staff	36/106 (34.0)	50/156 (32.1)	46/113 (40.7)
Telephone triage	40/109 (36.7)	57/159 (35.8)	64/120 (53.3)
Treating minor illness	43/105 (41.0)	49/156 (31.4)	83/124 (66.9)
Men's health	69/108 (63.9)	40/164 (24.4)	86/134 (64.2)
Treatment room sessions	70/107 (65.4)	73/162 (45.1)	36/124 (29.0)
Family planning	74/105 (70.5)	133/177 (75.1)	75/132 (56.8)
Breast awareness	79/106 (74.5)	123/175 (70.3)	42/125 (33.6)
Screening for new registrations	79/105 (75.2)	64/173 (37.0)	13/130 (10.0)
COPD	81/101 (80.2)	108/178 (60.7)	86/140 (61.4)
Diabetes	84/100 (84.0)	160/181 (88.4)	47/130 (36.2)
Asthma	84/100 (84.0)	166/186 (83.0)	50/136 (36.8)
Stroke	86/101 (85.1)	144/181 (79.6)	54/134 (40.3)
CHD	92/100 (92.0)	162/188 (86.2)	53/139 (38.1)
Health promotion	93/106 (87.7)	143/183 (78.1)	48/128 (37.5)
Travel immunizations	97/108 (89.9)	147/185 (79.5)	74/140 (52.9)
Cervical cytology	99/108 (91.7)	176/190 (92.6)	25/134 (18.7)

Continuing professional development over the previous three years reflected the increasing focus on chronic disease management, with 134 (67.0%) of respondents attending courses on diabetes, 92 (46.0%) CHD courses and 81 (40.5%) courses on stroke. 40 nurses (20.0%) had attended a nurse prescribing course, although 48 (24.0%) reported regularly prescribing medication; only 10 (5.0%) had attended a nurse practitioner course.

In-house training was common, with 149 (76.4%) participating in training activities in their practice in the previous 6 months and 126 (63.6%) participating in shared training sessions with the GPs in their practice in the previous 6 months.

### Professional support and career intentions

164 (86.3%) respondents had a Personal Development Plan and 173 (87.4%) had had a formal appraisal within the previous three years. However, only half the respondents felt their appraisal had been productive (85, 49.4%), with 70 (40.7%) finding it only a little productive and 17 (9.9%) reporting their appraisal to be unproductive. With regard to other professional support, 181 (91.4%) reported having someone they could discuss a clinical or professional problem with; 145 (74.0%) reported having someone they could discuss a personal problem with. When asked about isolation, however, 86 (43.7%) reported sometimes feeling isolated and 17 (8.6%) reported always feeling isolated. Finally, 30 nurses (15.5%) did not intend to continue working as a practice nurse in the coming 5 years. There was a significant association between age and the intention to leave practice nursing (Chi-square test for trend = 10.631, df = 1, p = 0.001), with 18 (60.0%) of those intending to leave aged 50 or more, however the other 12 (40.0%) were under 50 years.

### Association of isolation with demographic and workload variables

The factors associated with feeling isolated were examined more fully. Those replying "yes" or "sometimes" to the question of whether they ever felt isolated were grouped together and categorised as "isolated" with the others categorised as "non-isolated".

Those reporting feelings of isolation were more likely to be aged 40-49 and to be G Grade nurses, although these associations were not statistically significant (Table 3). Both groups had been practice nurses for a similar length of time (isolated group: median = 10.0 years (IQR: 4.0 - 15.0 years); non-isolated group: median = 11.0 years (IQR: 7.0 - 15.0 years); Mann-Whitney U test = 3875.5, p = 0.096). Isolated nurses worked in smaller practices (Table 3). The median practice list size for the isolated group was 5000 patients (IQR: 3000 - 7500); for the non-isolated group the median was 5500 patients (IQR: 4000 - 8500; Mann-Whitney U test = 3510.5, p = 0.016). Isolated nurses were more likely to work on their own or in smaller teams (Table 3). There was, however, no significant difference in either number of appointments or appointment times between the two groups (data not shown).

**Table 3:** Risk of feeling isolated by demographic and practice characteristics (Number (%)).

	Isolated nurses (n = 103)	Non-isolated nurses (n = 94)	p value
**Age categories (years)**			
20 - 39	18 (17.5)	24 (26.1)	Chi-square test for trend = 0.176, df = 1, p = 0.675.
40 - 49	57 (55.3)	39 (42.4)	
50 and above	28 (27.2)	29 (31.5)	
			
**Grade**			
D, E or F	7 (7.1)	8 (8.7)	Chi-square test for trend = 2.350, df = 1, p = 0.125.
G	79 (80.6)	61 (66.3)	
H	12 (12.2)	23 (25.0)	
			
**Practice list size**			
Up to 2000 patients	12 (12.1)	5 (5.6)	Chi-square test for trend = 5.107, df = 1, p = 0.024.
2001 - 4000	30 (30.3)	18 (20.2)	
4001 - 6000	24 (24.2)	29 (32.6)	
6001 - 8000	15 (15.2)	12 (13.5)	
8001 - 10,000	12 (12.1)	14 (15.7)	
Over 10,000 patients	6 (6.1)	11 (12.4)	
			
**Number of practice nurses in the team**			
1	40 (38.8)	21 (22.8)	Chi-square test for trend = 8.847, df = 1, p = 0.003.
2	44 (42.7)	39 (42.4)	
3 or more	19 (18.4)	32 (34.8)	

There were no significant differences in the qualifications/certificates obtained by both groups (data not shown), but only 64 (67.4%) of isolated nurses felt their training and qualifications were used to the full in their current job compared with 85 (92.4%) of non-isolated nurses (Fisher's Exact test, p < 0.001).

**Figure 1:**
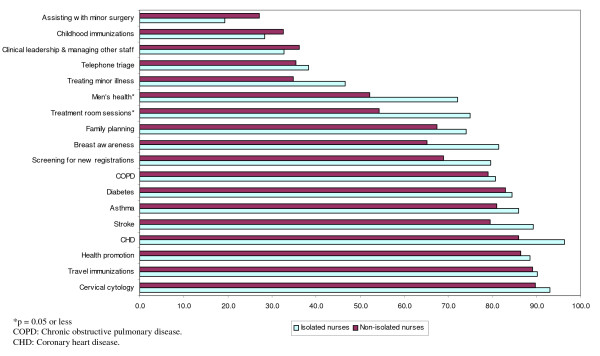
**Isolated and non-isolated practice nurse clinical activities (% of nurses reporting participating in each clinical activity)**.

There was little difference between the clinical activities undertaken by isolated and non-isolated nurses (Figure 1). However, a greater proportion of isolated nurses were involved in almost all of the listed clinical tasks. This difference was statistically significant for treatment room sessions (75.0% of isolated vs 54.3% of non-isolated: Fisher's Exact test, p = 0.038) and men's health (72.1% of isolated vs 52.2% of non-isolated: Fisher's Exact test, p = 0.043).

Isolated nurses were more likely to report needing more training (Table 4). This reached statistical significance for family planning, screening for new registrations, COPD, stroke, CHD and health promotion. Slightly fewer isolated nurses had attended recognised CPD courses across a range a clinical areas, but this was not statistically significant (data not shown). Isolated nurses were less likely to participate in within practice training sessions with other colleagues: 71.3% of isolated nurses vs 82.8% of non-isolated nurses (Fisher's Exact test, p = 0.063). Isolated nurses had had slightly fewer study days in the previous year, but this difference was not significant (isolated nurses: median of 4.0 days (IQR: 2.13 - 5.75); non-isolated nurses: median of 5.0 days (IQR: 3.00 - 9.25); Mann-Whitney U test = 3103.0, p = 0.087).

**Table 4:** Need for future training amongst isolated and non-isolated nurses carrying out the listed clinical activities (Number answering yes/Total number of responders (%)).

	Isolated nurses	Non-isolated nurses	Fisher's Exact test, p value
Assisting with minor surgery	15/55 (27.3)	15/54 (27.8)	1.000
Childhood immunizations	22/60 (36.7)	23/55 (41.8)	0.702
Clinical leadership & managing other staff	26/58 (44.8)	19/54 (35.2)	0.338
Telephone triage	39/64 (60.9)	24/55 (43.6)	0.068
Treating minor illness	48/67 (71.6)	34/56 (60.7)	0.250
Men's health	50/73 (68.5)	35/60 (58.3)	0.277
Treatment room sessions	23/67 (34.3)	12/56 (21.4)	0.160
Family planning	47/71 (66.2)	28/60 (46.7)	**0.033**
Breast awareness	25/67 (37.3)	17/57 (29.8)	0.448
Screening for new registrations	11/67 (16.4)	2/62 (3.2)	**0.017**
COPD	51/72 (70.8)	34/67 (50.7)	**0.023**
Diabetes	28/66 (42.4)	19/63 (30.2)	0.200
Asthma	31/69 (44.9)	19/66 (28.8)	0.074
Stroke	36/71 (50.7)	17/62 (27.4)	**0.008**
CHD	39/74 (52.7)	13/64 (20.3)	**< 0.001**
Health promotion	32/65 (49.2)	15/62 (24.2)	**0.006**
Travel immunizations	36/72 (50.0)	36/66 (54.5)	0.613
Cervical cytology	13/70 (18.6)	12/63 (19.0)	1.000

a. Adds to more than 200, as multiple responses were permitted.

Personal development plans were reported by 82.7% of isolated nurses and 90.1% of non-isolated nurses (Fisher's Exact test, p = 0.146). Both groups also reported similar levels of appraisal (84.5% isolated nurses vs 90.4% non-isolated nurses; Fisher's Exact test, p = 0.284). However, isolated nurses were more likely to report that their appraisal was unproductive (66.7% vs 33.3% non-isolated nurses, Fisher's Exact test, p < 0.001).

Fewer isolated nurses had access to someone with whom they could discuss a clinical or professional problem (85.4% isolated nurses vs 97.9% non-isolated nurses, Fisher's Exact test, p = 0.002) or a personal problem (62.7% isolated nurses vs 86.0% non-isolated nurses, Fisher's Exact test, p < 0.001). Only 77.3% of those who felt isolated planned to continue working as a practice nurse for the coming 5 years compared with 91.4% of non-isolated nurses (Fisher's Exact test, p = 0.009).

Within the Health Board area, there were opportunities for practice nurses to meet together. Approximately half of all practice nurses were able to attend these meetings. There was, however, no difference in attendance between nurses who felt isolated and those who did not (data not shown).

### Predictors of isolation

The results of the final binary logistic regression model are shown in Table 5. After accounting for the other variables, working alone was a highly significant predictor of isolation with single-handed nurses over 6-times more likely to report feeling isolated. Nurses working in teams of two were 3.5-times more likely to feel isolated. Training and qualifications being used to the full and having a productive appraisal both significantly reduced feelings of isolation, as did the intention to continue working as a practice nurse in the future, but this was not statistically significant.

**Table 5:** Association of nurse and practice characteristics with feeling isolated: Binary logistic regression model.

	Odds ratio (95% CI)	p value
**Number of practice nurses in the team (Reference group = 3 or more)**		
One	6.44 (2.13 to 19.46)	0.001
Two	3.49 (1.29 to 9.45)	0.014
		
**Training used to full (Reference group = no)**		
Yes	0.23 (0.08 to 0.67)	0.007
		
**Appraisal was productive (Reference group = no)**		
Yes	0.19 (0.08 to 0.43)	< 0.001
		
**Working as a practice nurse in 5 years time (Reference group = no)**		
Yes	0.33 (0.10 to 1.03)	0.056

## Discussion

Nurses working in UK general practice are an important part of the primary care workforce, particularly since the implementation of the new GMS contract [5,6,8]. In general, our findings agree with other national surveys conducted over the past 15 years, which showed that most practice nurses were aged 40 and over; most were Grade G nurses and that their workload covered a range of clinical activities, with immunization, cervical cytology, health promotion and chronic disease management clinics featuring prominently [24-27]. However, none of these surveys identified the feeling of isolation that was found here, nor its strong association with intentions to leave practice nursing. These nurses were older, more likely to be employed as Grade G nurses, worked in smaller practices and were either working alone or with one other nursing colleague. Although there was little difference between isolated and non-isolated nurses with respect to their qualifications, isolated nurses were more likely to feel that their qualifications were not being used to the full in their current job and were less likely to be planning to remain in practice nursing.

Isolated nurses were no busier than non-isolated nurses. Clinically, both groups had similar roles, although a greater proportion of isolated nurses participated in each clinical area - particularly in the provision of treatment room sessions, treating minor illness and men's health. More non-isolated nurses took part in activities related to clinical leadership and staff management and in assisting with minor surgery, suggesting that non-isolated nurses may take on more advanced roles within the practice. Although there may appear to be a contradiction in the findings that isolated nurses felt their skills were not used sufficiently, when they appeared to carrying out similar clinical tasks, there are potential explanations. Isolated nurses may be engaged in a wider range of activities, and so have less chance to develop in-depth knowledge in particular areas which could enhance their job satisfaction and sense of being needed in a team; alternatively, they may be feeling more uncertain in their role, particularly if they are covering many areas that they feel unprepared for. These issues could be explored in future studies.

A productive appraisal also appeared to mediate against feeling isolated. Participation in training activities within the practice and attendance at external practice nurse forum meetings was the same in both groups, suggesting (perhaps surprisingly) that such activities did not affect nurses' feelings of isolation. One potential explanation for this, however, might be that only around half of the respondents reported being able to attend such meetings in the first place.

Other studies have examined characteristics associated with intending to leave the profession, both in nursing[12,15,16] and general practice [28-30]. While factors such as age and workload were important, a key factor was job satisfaction. In some, this related to satisfaction with the job itself [28], while in others it related to wider factors, including dissatisfaction with promotion and training opportunities [12], changing requirements of the job and perceptions of being valued [15,16]. Feeling undervalued has been consistently reported by practice nurses since the advent of the 2004 GMS contract [5,6]. While we did not ask practice nurses directly about their level of satisfaction with their job, the finding that isolated nurses worked in smaller teams, felt that they did not use their training and qualifications to the fullest and had unproductive appraisals all point to potentially higher levels of dissatisfaction with their role today.

### Strengths and limitations

The survey targeted the entire population of practice nurses working in the Health Board area at that time. It achieved a response rate of 61%, lower than that obtained by Atkin et al in 1992 [24], and Caldow in 2000 [25], similar to that obtained by The Centre for Innovation in Primary Care in 2000 [26] and much higher than that obtained by the WiPP Snapshot Survey in 2006 [27]. It was also conducted at a time when practice nurses were coming to terms with the new GMS contract.

The lack of a practice nurse or practice identifier (as stipulated by the local ethics committee) meant that we could not gauge the representativeness of the responders in relation to the entire population, particularly in relation to the practice population served. In addition, as practice nurses are employees of UK general practitioners (themselves independent contractors), there is no centrally-held data on the demographics of this population. Responders were broadly similar to the characteristics reported for respondents in other, recent surveys of practice nurses [25-27]. Again, however, these surveys could not report on the characteristics of non-responders due to the lack of population-level data about this workforce. Based on respondents estimates of their practice list size, we can infer that there were more responses from nurses working in large practices (list size > 6000 patients: 37% of respondents' practices versus 26% of NHS Greater Glasgow's practices) and less from small practices (list size < 6000: 60% of respondents versus 75% of actual practices). Given the association between small practices and areas of socio-economic deprivation [31], this implies that there were fewer responses from nurses working in areas of deprivation. We also had no way of independently verifying the data, particularly in relation to workload and clinical activities.

This survey was conducted in late 2005, a time of great change within UK general practice as teams became used to the requirements of the new contract. Given the findings from more recent qualitative work, it is likely that nurses remain feeling isolated while dealing with an increasing workload associated with QOF. Nonetheless, it would be timely to repeat this work, and extend it to a national level, to clarify the current picture in relation to this important professional group.

The questionnaire used was one developed from that previously used within the health board and developed be reviewing the literature and in consultation with nursing colleagues within the board area. Although questionnaires have been used in other studies, these were not completely suitable either because of their content or their focus on hospital-based nursing [16,25,32,33]. However European studies of nurses' plans to leave hospital-based practice do confirm that issues such as perceived work ability, working conditions and support are important in nurses' views as to whether they wish to stay in nursing [16,32,33].

Finally, within the constraints of a self-completion questionnaire, it was not possibly to fully explore what nurses meant by isolation, nor whether this was a frequent or occasional feeling. Free text comments indicated a number of reasons for isolation, including that of working alone within a practice and lack of opportunities for clinical teaching and supervision. In order to fully explore this issue, further qualitative work is recommended.

## Conclusions

Finding solutions to nurses' reports of isolation is of paramount importance, not only for practice nurses as a profession but also for the future development of general practice. Recognition of the role of practice nurses, nationally agreed terms and conditions and more multi-professional training initiatives have been suggested [34,35]. One explanation may be that nurses who report feeling isolated are also, in themselves, less likely to seek opportunities for training and support. However, our findings show that isolated nurses had similar amounts of study leave as non-isolated nurses and attended similar numbers of external practice support meetings. This suggests that while area-based initiatives are important, many of the solutions lie within general practices themselves. Research shows that job satisfaction, and presumably lack of isolation, is highest in practices with a good team climate, irrespective of the number of practice staff [36,37]. Therefore, we suggest that primary care organisations target their effort on supporting and building the team environment within general practices, regardless of size or staff composition, and that improving conditions for one group of staff should have a positive effect on all staff.

## Competing interests

The authors declare that they have no competing interests.

## Authors' contributions

CO, HJ and GW designed the study; HJ conducted the study as part of his PhD degree and conducted the main analyses, with COD and GW supervising. COD conducted additional analyses and drafted the manuscript; all authors commented on the drafts, saw and approved the final version.

## Pre-publication history

The pre-publication history for this paper can be accessed here:

http://www.biomedcentral.com/1472-6955/9/2/prepub

## Supplementary Material

Additional file 1 Practice nurse survey. Click here for file
