# Supplementary material for: Practice nurses' workload, career intentions and the impact of professional isolation: A cross-sectional survey
Source: BMC Nurs. 2010 Jan 25;9:2. doi: 10.1186/1472-6955-9-2 (PMC2823612; doi:10.1186/1472-6955-9-2)
Supplement: Additional file 1 — Practice nurse survey. [file 1472-6955-9-2-S1.DOC]

# PRACTICE NURSE SURVEY

### PRIMARY CARE DIVISION

### GENERAL PRACTICE & PRIMARY CARE, UNIVERSITY OF GLASGOW.

Please tick in the boxes as appropriate:

1a) Are you: Full time

Part time

1b) Please indicate how many hours you are contracted to work per week

1c) In addition, do you regularly work additional (overtime) hours?

Yes No

1d) If yes; how many additional hours per week on average

2) What is your present job title? Please select one of the following:

|  | Practice nurse |
| --- | --- |
|  | Senior Practice Nurse |
|  | Practice Nurse manager |
|  | Nurse Practitioner |
|  | Staff Nurse |
| Other | Please state:…………………………… |

3) Approximately how many patients are on the practice list?

4a) How many practice nurses do you have in the practice?

4b) Are the practice nurses, within your practice, employed as?

|  | A structured team |
| --- | --- |
|  | A group of individuals with no leader |
|  | Not applicable |

5a) Is there a practice nurse leader within your practice?

##### Yes No

##### 5b) If yes, is this seniority recognized by him/her being on a different staff grade?

###### Yes No Not applicable

5c) Do any of the practice’s district nursing team hold treatment room sessions?

Yes No

**About yourself:**

6) What is your age? 20 – 29 50 – 59

30 – 39 60 & above

40 – 49 Declined to answer.

7) What is your current staff grade?

D F H Other

E G N/A

8a) How long have you been a practice nurse?

Less than 1 year

| Years |
| --- |

8b) How long have you been with your present practice?

Less than 1 year

| Years |
| --- |

9) What position did you hold before becoming a Practice Nurse?

|  |
| --- |

10a) What qualification do you hold? (tick all that apply):

EN RGN/SRN SCM/SM

RMN DN HV

Nursing degree Practice nurse’s certificate

Specialist Nurse in General Practice Masters degree

| Other | Please state: |
| --- | --- |

10b) Do you think that your training and qualifications are used to the full in your

current job? Yes No

11a) Initially, what was your main reason for choosing to become a practice nurse?

|  | The job, itself |
| --- | --- |
|  | I saw it as a career |
|  | The hours suited my commitments |
|  | The autonomy |
| Another | Please state: |

11b) Looking a head, would you envisage to continue work as a practice nurse for

the coming 5 years? Yes No

12a) Do you work in clinics with appointment system

Yes No

12b) If yes, how many appointment slots do you have per day?

12c) How long, on average, are your appointment slots?

| Minutes |
| --- |

13) What is the scope of your work and training? Please use the following table:

|  | ***Hours***  ***per***  ***week.*** | Have you had any specialized training in this role? | | ***Do you feel***  ***you need***  ***more training?*** | |
| --- | --- | --- | --- | --- | --- |
| 1. **General** |  | **Yes** | **No** | **Yes** | **No** |
| **Cervical Cytology** |  | Yes | No | Yes | No |
| Breast awareness |  | Yes | No | Yes | No |
| Family planning |  | Yes | No | Yes | No |
| Health promotion |  | Yes | No | Yes | No |
| Travel Immunizations |  | Yes | No | Yes | No |
| Childhood Immunizations |  | Yes | No | Yes | No |
| Men’s health |  | Yes | No | Yes | No |
| Telephone triage |  | Yes | No | Yes | No |
| Treatment room sessions |  | Yes | No | Yes | No |
| **Treating Minor Illnesses** |  | Yes | No | Yes | No |
| **Screening for new registrations** |  | Yes | No | Yes | No |
| **Clinical leadership & managing other staff** |  | Yes | No | Yes | No |
| **Assisting with minor surgery** |  | Yes | No | Yes | No |
| 1. **Chronic Disease Management** |  |  |  |  |  |
| **Diabetes** |  | Yes | No | Yes | No |
| **Asthma** |  | Yes | No | Yes | No |
| **COPD** |  | Yes | No | Yes | No |
| **CHD** |  | Yes | No | Yes | No |
| **Stroke** |  | Yes | No | Yes | No |
| 1. **Others; Please state** |  | **Yes** | **No** | **Yes** | **No** |
|  |  |  |  |  |  |
|  |  |  |  |  |  |
|  |  |  |  |  |  |

14a) Are you involved in any aspect of audit? Yes No

14b) If yes, have you had training in audit?

Yes Some training None at all

14c) Do you require training in audit? Yes No

14d) Are you involved in any aspect of Clinical Research?

Yes No

15a) How are your Holiday / other absences covered?

|  | Colleagues increase hours to cover |
| --- | --- |
|  | Your work commitment are cancelled |
|  | GGNHS Practice Nurse Locum Service |
| Other | please state: |

15b) Do you find the locum service satisfactory? Yes No

16) Do you undertake sessions with the GGNHS Practice Nurse Performers List

(Locum List)? Yes No

## Training issues:

17a) In the last 3 years, Have you undertaken, or are currently undertaking, any recognized (E.g. with a certificate) courses relating to your practice nurse work? Please tick as appropriate from the following box:

|  | **Course** | Yes | **No** |
| --- | --- | --- | --- |
| 1 | **Asthma** |  |  |
| 2 | Diabetes |  |  |
| 3 | Epilepsy |  |  |
| 4 | Marie Curie breast and cervical screening |  |  |
| 5 | Family planning |  |  |
| 6 | Triage |  |  |
| 7 | Stroke |  |  |
| 8 | **Multiple Sclerosis** |  |  |
| 9 | COPD |  |  |
| 10 | CHD |  |  |
| 11 | Nurse Practitioner |  |  |
| 12 | Nurse prescribing |  |  |
| 13 | **Other (please state)** | | |

17b) I haven’t done any courses in the last 3 years

18a) Do you regularly undertake nurse prescribing?

Yes No

18b) Do you have a nurse-prescribing certificate/qualification?

Yes No

18c) If the answer is no, does your work involve prescribing medications for your patients with back up from the GP?

Yes No

18d) Do you think that nurses should have an independent role in prescribing new medications for chronic diseases?

Yes No

18e) Do you think nurses should have an independent role in prescribing for an agreed list of conditions?

Yes No

19a) Do you have the opportunity for Continuing Professional Development (CPD) activities?

Yes No

If No why?

19b) What CPD you would like to see in place?

**Training support**

20a) How many study days did you have last year?

20b) Is it easy to attend study days?

Yes No

20c) What inhibits you from attending study days?

|  | Financial reasons |
| --- | --- |
|  | Getting time off work |
|  | The problem of travelling long distance to courses |
| Other | please state: |

20d) Who decides what study days you attend?

|  | GP |
| --- | --- |
|  | Practice Manager |
|  | Lead Practice nurse |
| Other | please state: |

21) Training courses: please answer the related questions in the following box:

| Training |  |  |  |
| --- | --- | --- | --- |
| a. Your training time is / was:  1. Part of your normally paid working commitment?  OR  2. Additional hours to your normally paid working commitment? | Yes | Some times | No |
|  | Yes | Some times | No |
| b. Are your course fees paid for you | Yes | Some times | No |

22a) Did you participate in any shared training / continuing education sessions with

doctors in the last 6 months? Yes No

22b) If yes, the number of sessions

23a) Have you participated in regular training activities at your practice in the last 6 months?

|  | With the nursing colleagues only. |
| --- | --- |
|  | With GP colleagues |
|  | With both GPs and PNs |
|  | No |

23b) Do you have In-Service Continuing Training/Education activities at your

practice? Yes No

23c) Any other comments about in service training

24a) Do you have a Personal Development Plan?

Yes No

24b) Have you had a formal appraisal in the last 3 years?

Yes No

N/A (e.g., too recently in post)

24c) If yes, who was it with?

|  | Practice Manager |
| --- | --- |
|  | Lead Practice nurse |
|  | GP |

24d) If yes, was it productive?

Yes A little No

25a) A lot of practices employ Health Care Support Workers (HCSW) for what could be described as ‘practice nurse’ duties. Does your practice employ

any? Yes No

25b) If yes, who are they? Receptionist

| Other | Please state: |
| --- | --- |

25c) If yes, what do they do? Phlebotomy

Blood Pressure

Height and Weight

Urinalysis

New Patient Medicals

| Other | Please state: |
| --- | --- |

25d) What sort of training has this member of staff had?

|  | Glasgow Caledonian University course |
| --- | --- |
|  | Bradford Distance Learning course |
|  | In-house training only |
|  | I do not Know |

26a) Do you act as mentor for the Health Care Support Workers (HCSW)?

Yes No

26b) Have you had training in mentorship?

Yes No

**Communication:**

27a) Do you have access to someone with whom you could discuss for example:

1. A clinical / professional problem

Yes May be / Unsure No

1. Personnel type problem

Yes May be / Unsure No

27b) Do you ever feel isolated (or alone, lacking opportunities for clinical supervision) in your work situation?

Yes Sometimes No

28a) Do you have the opportunity to be part of clinical supervision sessions?

|  | Yes, regularly |
| --- | --- |
|  | Sometimes |
|  | Rarely |
|  | Never |

28b) If you do not take part in clinical supervision, why not?

29a) Are you aware of the Glasgow local practice nurse group?

Yes No

29b) Do you have the opportunity to attend it’s meetings?

Yes Rarely Never

29c) Do you attend your LHCC practice nurse meetings?

Yes Rarely Never

29d) Do you find the LHCC practice nurse group meetings with the Practice Nurse

Advisor advantageous? Yes No

29e) Comments

30a) What prevents you from attending practice nurse meetings in general?

|  | Time constraints (eg, clinics) |
| --- | --- |
|  | Location of meetings |
|  | Unaware – no information |
|  | Content of meetings doesn’t appeal |
| Other | Please state |

30b) If you don’t attend due to the content of the practice nurse meetings, what would you like to see in the meetings that would encourage you to come?

31a) Do you receive information from the practice nurse advisor?

Yes Sometimes No

31b) Do you receive information from GGNHS Primary Care Division?

Yes Sometimes No

32a) Would you prefer information to come to you via:

Email Paper

32b) Do you have ready access at work to email?

Yes No

1. Any other comments regarding support issues:

|  |
| --- |

**General:**

34) Any general comments:_________________________________________

_________________________________________

**Thank you for taking the time to complete this questionnaire.**

**You will receive feedback once all the data has been collated.**
